# Effects of injection of hydrolysis plasma protein solution on the antioxidant properties in porcine *M. Longissimus Lumborum*

**DOI:** 10.1186/s40781-016-0111-7

**Published:** 2016-08-17

**Authors:** Hyun-Woo Seo, Jin-Kyu Seo, Han-Sul Yang

**Affiliations:** 1 National Institute of Animal Science, RDA, Wanju, 55365 South Korea; 2 Division of Applied Life Science Graduate School (BK21 plus), Institute of Agriculture and Life Science, Gyeongsang National University, Jinju, 52828 South Korea

**Keywords:** Hydrolysis plasma proteins, Shear force, Lipid peroxidation, Meat quality

## Abstract

**Background:**

Plasma protein hydrolysates have been shown to possess antioxidant activity. However, no report has yet to examine the antioxidant effects of injection of plasma protein hydrolysates on meat quality. Therefore, in this study, the effects of injection of hydrolysis plasma protein solution on meat quality and storability were investigated in porcine *M. longissimus lumborum*.

**Methods:**

Twelve pigs were randomly selected at a commercial slaughter plant and harvested. Dissected loins were injected with one of five solutions: C- control (untreated), T1- 10 mM phosphate buffer solution (PBS), T2- 10 mM PBS with 0.01 % butylated hydroxytoluene, T3- 10 mM PBS with 5 % plasma proteins, and T4- 10 mM PBS with 5 % hydrolysis plasma proteins.

**Results:**

T3 and T4 induced greater reduction in protein content of the loin muscle than other treatments. T2 resulted in the lowest pH as well as highest cooking loss. After a storage period of 3-7 days, both lightness and redness of meat were unaffected by all injection treatments. However, yellowness was significantly elevated by treatment with T4 relative to the control. T4 also resulted in the lowest shear force (a measure of meat toughness), suggesting improvement of texture or tenderness. Further, T4 resulted in the most stable TBARS values during storage, indicating that this treatment might retard rancidity in meat.

**Conclusion:**

Injection of porcine *M. longissimus lumborum* with hydrolysis plasma protein solution could improve overall pork quality, including tenderness and storability.

## Background

Addition of marinade solutions to pork and other meat products to enhance quality is a well-established practice in many countries [1, 2]. A combination of salt and phosphate is commonly used to induce synergistic effects in meat tissue [3–5]. This procedure not only improves juiciness and tenderness but also increases the weight of the saleable product, caused by the retention of added water. The concentration of the additive needs to be such that tenderness and juiciness are improved but flavor and color are not adversely affected and the meat is not over-tenderized. The mechanism responsible for increased tenderness and juiciness is linked to increased water retention, and consequent swelling, of myofibrils in the meat [6].

The relative efficiency of marination for improving juiciness and tenderness in meat has been established based on sensory evaluation [7]. In a prior study, Killefer [8] injected pork loins 1 h after the animal was slaughtered with a solution of citrate, phosphate, and salt or a solution of only phosphate and salt (control). In their results, increased ultimate pH values, improved color, and decreased cooking loss and shear force values were reported for the treated meat compared to the controls. Sodium citrate has been used as a glycolytic inhibitor in beef muscle to improve tenderness [9]. It has been hypothesized that the pH increase resulting from glycolytic inhibition creates an environment in which protein-denaturing calpains are likely more active. Phosphate injection was originally developed to reduce the the sodium content of processed meats such as ham but has been gradually incorporated into fresh meats as well in order to improve their tenderness and juiciness [10]. Phosphate ‘enhancement’ is now commonly used in the pork industry to increase pH and improve pork quality attributes. Although phosphate injection may increase saltiness and decrease the shelf life of meat [11], routine use of this method in industry necessitates its inclusion in a study that compares various new technologies for improving pork quality.

The most common synthetic antioxidants, such as butylated hydroxyanisole (BHA) and butylated hydroxytoluene (BHT), have been widely used for many years to delay lipid oxidation and extend shelf-life of meat [12]. However, concerns about the long-term safety and negative consumer perception of synthetic antioxidants have led to an increasing demand for natural antioxidants in meat and meat products [13]. It has been reported that some protein and enzymatic hydrolysates of meat and meat by-products exert antioxidant effects in food systems [14]. For instance, plasma protein hydrolysates have been shown to possess antioxidant activity [15]. However, no information on the effect of injection of antioxidants on meat quality has been published.

Therefore, the purpose of this study was to investigate the effects of plasma protein injection into pre-rigor porcine *M. longissimus lumborum* on ultimate pork quality characteristics such as color, muscle pH, shear force, protein solubility, and rancidity during cold storage.

## Methods

### Preparation of non-hydrolysis and hydrolysis bovine plasma protein

To prepare plasma proteins (PP), from cattle blood plasma, 0.5 N ethylenediaminetetraacetic acid (EDTA) as an anticoagulated was added to fresh cattle blood at a ratio 1:9 (v/v), mixed well, and placed immediately on ice for 30 min. Samples were centrifuged by a refrigeration centrifuge (SUPRA 25 K, Hanil Science, Korea) at 14,000 *g* for 15 min at 4 °C. The plasma powders were freeze-dried (Clean van 8B Freeze-Dryer, BioTron, Inc., Korea), pulverized, placed in sealed bags, and stored at 4 °C.

To prepare plasma protein hydrolysate (PPH), PP solution [5 % w/v 10 mM sodium phosphate buffer (pH 7.0)] was heat-pretreated (90 °C, 5 min) and then hydrolyzed with Alcalase. The enzyme to substrate ratio (E/S) was 2:100 (g/g). The pH of PP solution was adjusted to the optimal value for Alcalase (pH 8.32) before hydrolysis and was readjusted to the optimal value with 1 M NaOH every 15 min during hydrolysis. Hydrolysates were produced by varying the hydrolyzed time to 338 min and hydrolyzed temperature 54 °C. After hydrolysis, the pH of the solution was brought to 7.0 and the solution was then heated at 95 °C for 5 min to inactivate the enzyme. Degree hydrolysis (DH) was determined by assaying free amino groups with 2, 4, 6-trinitrobenzenesulfonic acid (TNBS) according to Alder-Nissen [16]. The DH of hydrolyzed PP was 18.8 %.

### Preparation of samples

Approximately 50 min post-mortem, dissected loins were assigned to injection treatments as follows: C- control (untreated), T1- 10 mM phosphate buffer solution (PBS) (pH 7.0), T2- 10 mM PBS with 0.01 % butylated hydroxytoluene (BHT), T3- 10 mM PBS with 5 % plasma proteins, and T4 10 mM PBS with 5 % hydrolysis plasma proteins. Before injection, skin was sliced perpendicular to the length of the loin, at approximately 3-cm intervals, in order to allow the injection needle to penetrate the muscle. Solutions were injected at room temperature. A hand-held injector and 10-cm needles were used to inject the experimental solutions. After injection, pump percentage was calculated. It was assumed that the loin constituted 10 % of the total weight of the side, and absorbed all of the injected solution. Pigs were stunned by using both an electric stunning wand and a captive bolt stunner. after stunning, pigs were exsanguinated and harvested according to normal procedures; the procedure was approved by the institutional Animal Care and Use Committee. After the carcasses and washed, each side was weighed.

### Proximate chemical composition analysis

The proximate chemical compositions of the marinated samples were determined following standard procedures prescribed by the Association of Official Analytical Chemists [17]. Moisture, crude protein, fat, and ash contents were determined using the oven, Folch et al*.* [18], Kjedahl, and dry ashing methods, respectively.

### pH measurement

Approximately 3 g of each meat sample were weighed out, and distilled water was added. A slurry was made out of the meat and distilled water using a homogenizer (Ultra Turrax T25D, IKA, Germany). The pH of each slurry sample was measured, in triplicate, using a digital pH meter (MP230, Mettler Toledo, Switzerland).

### Cooking loss

Weights of the uncooked and cooked samples were recorded (as per Boles and Swan, [19]), and cooking yield was calculated as follows:

$$ \mathrm{Cooking}\ \mathrm{loss}\ \left(\%\right) = \left(\mathrm{cooked}\ \mathrm{weight}/\mathrm{uncooked}\ \mathrm{weight}\right) \times 100 $$

Loss due to cooking was determined.

### Color evaluation

The internal color (International Commission on Illumination L^*^ (lightness), a^*^ (redness), and b^*^ (yellowness)) of the injected porcine *M. longissimus lumborum* samples were measured using a Minolta Chromameter (Minolta CR 301, Tokyo, Japan) and standardized with a white calibration plate (*Y* = 93.5; *x* = 0.3132; *y* = 0.3198). Internal color was measured at three random locations of the sample surface, and the mean of these values was used in statistical analyses.

### Myoglobin content measurement

The concentration of myoglobin forms was determined according to the method described by Krzywicki [20]. Myoglobin was extracted from meat samples using phosphate buffer with a pH of 6.8 and ionic strength of 0.04. The final ratio of buffer to meat in the extracts was 5:1. The absorbance levels of the extract at four wavelengths (572, 565, 545, and 525 nm) was measured using a spectrophotometer (8453 UV-visible Agilent Co. U.S.A.) The linear relationship between absorbance and myoglobin concentration was checked for all wavelengths used. The concentration of myoglobin forms (MetMb) was calculated as

$$ \mathrm{MetMb} = -2.514{\mathrm{R}}_1 + 0.777{\mathrm{R}}_2 + 0.800{\mathrm{R}}_3 + 1.098 $$

where R_1_, R2, and R3 are the absorbance ratios A_572_/A_525_, A_565_/A_525_, and A_545_/A_525_ respectively.

### Protein solubility measurement

In order to determine the solubility of sarcoplasmic and total (sarcoplasmic + myofibrillar) proteins, two extractions were conducted. First, sarcoplasmic proteins were extracted with 10 mL of ice-cold 25 mM potassium phosphate buffer (*pH* = 7.2), which was added to each of the quadruplicate 1-g muscle samples [21]. The samples were then cut up with scissors, homogenized on ice using a Polytron on the lowest setting (3 × 4-second bursts to minimize protein denaturation through heating), and left on a shaker at 4 °C overnight. Next, the samples were centrifuged at 1,500 × *g* for 20 min and the protein concentrations of the supernatants were determined by the biuret method, using bovine serum albumin as the standard. Second, total protein was extracted with 20 mL of ice-cold 1.1 M potassium iodide solution in a 0.1 M phosphate buffer (*pH* = 7.2) which was added to duplicate 1 g samples. Homogenization, shaking, centrifugation, and protein determination of the samples were performed as described for sarcoplasmic proteins. Myofibrillar protein concentration was calculated as the difference between total and sarcoplasmic protein concentrations.

### Shear force analysis

Cooked meat samples were allowed to cool to 25 °C, after which three 1.27 cm core samples, oriented parallel to the muscle fiber structure of the meat, were excised. Warner-Braztler shear force, perpendicular to the muscle fiber orientation, was determined for each core using an Instron Universal Testing Machine (Model 1000) with a load cell of 50 kg and a chart speed of 100 mm/min.

### Lipid oxidation

The thiobarbituric acid-reactive substance (TBARS) contents of the samples, from each treatment, were determined using the TBA distillation procedure modified by Burge and Aust [22]. Five-gram samples were weighed and homogenized using a homogenizer (Ultra Turrax T25D, IKA, Germany). The homogenate of the samples was transferred to a disposable test tube, into which 10 % butylated hydroxyanisole, and thiobarbituric acid/trichloroacetic acid (TBA/TCA) solution were added. The sample was mixed using a vortex mixer, and then incubated in a boiling water bath for the development of color. After cooling, supernatant solution was determined at 531 nm. The TBARS values were expressed as the number of milligrams of malondialdehyde per kilogram of sample.

### Statistical analysis

Data were analyzed by ANOVA test and Duncan’s multiple comparison was applied to test the significance of differences between groups. Statistical Analysis Systems (SAS, [23]) was used for analyzing data.

## Results and Discussion

### Proximate chemical composition analysis

Injection of hydrolysis plasma proteins (T4) and simple plasma proteins (T3) decreased the protein contents of the treated samples (*p* < 0.05; Table 1). However, injection of plasma proteins into porcine *M. longissimus lumborum* tissue had no effect on fat and ash contents of the samples. The achieved injection gains were close to the target value of 10 % of muscle weight, likely due to significant increases in moisture contents of the samples in response to all treatments (*p* < 0.05; Table 1).

**Table 1:** Effects of injection with plasma protein solution on proximate composition (%) in porcine *longissimus* muscle

Treatments^1)^	Moisture (%)	Fat (%)	Protein (%)	Ash (%)
C	71.82 ± 0.34^C^	4.85 ± 0.37	19.78 ± 0.10^A^	1.03 ± 0.01
T1	74.22 ± 0.45^B^	4.37 ± 0.30	19.73 ± 0.10^A^	1.02 ± 0.04
T2	75.35 ± 0.34^A^	4.95 ± 0.08	19.38 ± 0.32^A^	1.06 ± 0.04
T3	73.95 ± 0.29^B^	4.91 ± 0.24	18.45 ± 0.21^B^	1.06 ± 0.02
T4	75.17 ± 0.15^A^	4.81 ± 0.22	17.38 ± 0.01^C^	1.06 ± 0.02

^A–C^Means with different superscripts in the same column significantly differ at *p* < 0.05

^1^C-control, T1-injection with 10 mM sodium phosphate buffer (*pH* = 7.0) at 10 %, T2-injection with 10 mM sodium phosphate buffer (*pH* 7.0) with 0.01 % BHT at 10 %, T3-injection with 10 mM sodium phosphate buffer (pH 7.0) with 5 % plasma protein at 10 %, and T4-injection with 10 mM sodium phosphate buffer (*pH* 7.0) with 5 % hydrolysis plasma protein at 10 %

### pH and cooking loss

The pH values of T1, T2, and T3 were significantly lower than those of T4 and the control (*p* < 0.05; Table 2). However, during cold storage, samples injected with the hydrolysis and non-hydrolysis plasma protein solutions (T3 and T4) showed decreased pH values (*p* < 0.05). Contrary to our findings, pH has been shown to increase in samples treated with phosphate and bicarbonate [5, 24, 25].

**Table 2:** Effects of injection with plasma protein solution on pH and cooking loss (%) in porcine *longissimus* muscle, during cold storage

Treatments^1)^		Storage (days)		
		1	3	7
pH	C	5.67 ± 0.02^Aa^	5.68 ± 0.02^Aa^	5.64 ± 0.01^Ab^
	T1	5.54 ± 0.04^B^	5.53 ± 0.03^B^	5.48 ± 0.02^B^
	T2	5.45 ± 0.02^C^	5.47 ± 0.01^C^	5.42 ± 0.05^C^
	T3	5.54 ± 0.01^Ba^	5.45 ± 0.01^Cb^	5.46 ± 0.01^BCb^
	T4	5.69 ± 0.03^Aa^	5.71 ± 0.02^Aa^	5.63 ± 0.01^Ab^
Cooking loss (%)	C	40.21 ± 0.26^Ca^	38.22 ± 0.14^Bc^	38.95 ± 0.82^Bab^
	T1	46.91 ± 1.64^A^	43.65 ± 0.48^A^	41.72 ± 2.26^AB^
	T2	45.96 ± 1.64^AB^	44.13 ± 0.35^A^	44.00 ± 0.86^A^
	T3	41.75 ± 2.17^BC^	39.25 ± 2.64^B^	40.95 ± 0.26^AB^
	T4	41.89 ± 2.68^BC^	43.41 ± 1.67^A^	42.19 ± 0.91^AB^

^A–C^Means with different superscripts in the same column significantly differ at *p* < 0.05

^a–c^Means with different superscripts in the same row significantly differ at *p* < 0.05

^1^C-control, T1-injection with 10 mM sodium phosphate buffer (*pH* 7.0) at 10 %, T2-injection with 10 mM sodium phosphate buffer (*pH* = 7.0) and 0.01 % BHT at 10 %, T3-injection with 10 mM sodium phosphate buffer (*pH* = 7.0) and 5 % plasma protein at 10 %, and T4-injection with 10 mM sodium phosphate buffer (*pH* = 7.0) and 5 % hydrolysis plasma protein at 10 %

Quantification of expressible moisture (EM), a measure of the water-holding capacity (WHC) of meat, involves the use of force to expel water from the meat [26, 27]. Therefore, lower EM values coincide with increased breaking force values [28]. Myofibrillar proteins, myosin, actin, and, to some extent, tropomyosin are the main water-binding components of muscular tissue [27]. Denatured or precipitated sarcoplasmic proteins bound to myofibrils play an important role in decreasing the WHC of meat [27, 28].

Cooking loss of samples treated with the experimental solutions was significantly (*p* < 0.05) higher than those of samples treated with the control, during cold storage (Table 2). Improvements in WHC, observed in meat treated with sodium bicarbonate, may be attributed to increases in muscle pH and ionic strength [29]. Ionic strength may be related to the amount of ions in solution; sodium bicarbonate increases the number of ions, which react with proteins, as well as hydration. However, the treatments in this study failed to improve the WHC of the meat samples. During cold storage, cooking loss of samples injected with the hydrolysis and non-hydrolysis plasma protein solutions was lower than those of samples treated with BHT solution.

### Color

After 1 day, the lightness and redness of meat injected with plasma protein solution were higher than those of meat injected with hydrolysis plasma protein solution. Yellowness induced by the treatments significantly decreased (*p* < 0.05) with storage time, although this decrease was not observed in meat treated with the control solution. During cold storage, the yellowness of meat injected with hydrolysis plasma protein solution was significantly higher than that of meat injected with the other solutions (*p* < 0.05; Table 3).

**Table 3:** Effects of injection with plasma protein solution on CIE L*, a*, and b* in porcine *longissimus* muscle, during cold storage

Treatments^1)^		Storage (days)		
		1	3	7
L* (lightness)	C	58.29 ± 3.02^AB^	57.84 ± 2.86	57.65 ± 1.54
	T1	58.39 ± 2.01^AB^	58.90 ± 3.56	58.42 ± 2.63
	T2	56.53 ± 3.11^B^	56.46 ± 3.86	56.91 ± 2.60
	T3	60.04 ± 0.87^A^	56.88 ± 3.22	57.22 ± 2.48
	T4	58.18 ± 2.32^AB^	59.89 ± 3.72	57.94 ± 4.95
a* (redness)	C	7.77 ± 0.78^BC^	7.46 ± 0.54	7.32 ± 0.79
	T1	8.56 ± 0.46^ABa^	7.60 ± 1.12^ab^	6.93 ± 0.90^b^
	T2	8.95 ± 1.04^Aa^	7.25 ± 1.10^b^	7.20 ± 0.55^b^
	T3	7.33 ± 0.54^C^	7.07 ± 1.38	6.46 ± 0.89
	T4	8.40 ± 0.49^AB^	7.43 ± 0.93	7.44 ± 1.41
b* (yellowness)	C	6.68 ± 1.20^C^	7.35 ± 0.68^B^	6.65 ± 0.65^B^
	T1	7.88 ± 0.38^ABa^	7.30 ± 0.77^Bab^	6.60 ± 0.50^Bb^
	T2	7.77 ± 0.27^ABa^	6.49 ± 0.36^Cb^	6.50 ± 0.40^Bb^
	T3	7.10 ± 0.64^BCa^	6.85 ± 0.42^BCa^	5.93 ± 0.60^Bb^
	T4	8.46 ± 0.18^Aa^	8.29 ± 0.34^Aa^	7.61 ± 0.54^Ab^

^A–C^Means with different superscripts in the same column significantly differ at *p* < 0.05

^a–b^Means with different superscripts in the same row significantly differ at *p* < 0.05

^1^C-control, T1-injection with 10 mM sodium phosphate buffer (*pH* = 7.0) at 10 %, T2-injection with 10 mM sodium phosphate buffer (*pH* = 7.0) and 0.01 % BHT at 10 %, T3-injection with 10 mM sodium phosphate buffer (*pH* = 7.0) with 5 % plasma protein at 10 %, and T4-injection with 10 mM sodium phosphate buffer (*pH* = 7.0) and 5 % hydrolysis plasma protein at 10 %

This color change in pork loin was expected based on a previously established strong positive relationship between color of pork and pH [30, 31]. PSE and DFD pork differ from normal pork in terms of physiological and biochemical characteristics. The unusual pH and WHC of the PSE and DFD muscles lead to unusual meat colors [32]. In this study, the effects of hydrolysis plasma protein solution injection on muscle pH were dramatic. As the ultimate pH level of the plasma protein solution treated samples was not significantly different (Table 2), the higher a* and b* values can be directly attributed to the injection of hydrolysis plasma protein into pork.

Meat color is one of the most important factors influencing the quality and consumer preferences related to meat, and is considered as an indicator of meat freshness and ‘doneness’ (i.e., how well a meat is cooked) [33]. In measuring bloom on the surface of muscles, Brewer et al*.* [34] reported that the L^*^, a^*^, and b^*^ values were most correlated to the visual determination of muscle surface pinkness (*r* = -0.67 to -0.80). Lindahl et al*.* [35] reported that heme pigment and metmyoglobin contents are only slightly correlated with peak L^*^ values (*r* = 0.35–0.45). Furthermore, heme pigment and metmyoglobin contents were less correlated with b^*^ than with a^*^ values (*r* = 0.40 and 0.50, respectively). Generally, changes in L^*^ values (lightness) over the period of retail display were very subtle [36]. The oxymyoglobin and myoglobin fractions in meat were found to be the most important factors related to variations in b^*^ values [35]. According to Lindahl et al*.* [35] observed decreases in b^*^ suggest that the color of pork became less yellow because browning reactions (lower ratio of myoglobin to oxymyoglobin) in cooked meat were fewer.

### Changes in metmyoglobin (MetMb)

During cold storage, although the MetMb content (%) of meat treated with the experimental solutions was lower than that of meat treated with the control, all meat samples showed increased MetMb content (Table 4). Hydrolysis plasma proteins significantly decreased MetMb content (%) during cold storage. Meat color is influenced by many factors, including the concentration of heme pigments, chemical state of myoglobin (Mb), and physical characteristics. Of these factors, myoglobin, a sarcoplasmic protein, is primarily responsible for meat color. Myoglobin is a heme protein that exists in three forms deoxy-myoglobin (DexyMb), oxy-myoglobin (OxyMb), and MetMb [37]). Upon exposure to air, Mb combines with oxygen to form ferrous OxyMb, which is bright red in color; this bright red color is generally interpreted by consumers as an indication of the freshness of meat. However, extended contact of Mb with oxygen leads to the formation of the oxidized form, ferric MetMb, which is brown and unattractive. During cold storage, the rate of MetMb accumulation on the surface of meat is governed by many intrinsic factors (e.g., pH, muscle metabolic type, animal age, breed, sex, and diet), extrinsic factors (e.g., temperature, oxygen availability, type of lighting, surface microbial growth, and type of packing) or combinations of the two [38]. It has been widely accepted that beef muscles exhibit a wide range of color stability during cold storage [39]. However, the mechanism by which MetMb accumulation influences color is yet to be fully understood. One proposed mechanism for MetMb accumulation is the simultaneous decrease in a^*^ values [40].

**Table 4:** Effects of injection with plasma protein solution on metmyoglobin percentage of porcine *longissimus* muscle, during cold storage

Treatments^1)^	Storage (days)		
	1	3	7
C	4.92 ± 0.04^Ac^	7.52 ± 0.04^Ab^	11.54 ± 0.07^Aa^
T1	4.65 ± 0.07^Bc^	6.60 ± 0.02^Bb^	9.87 ± 0.03^Ba^
T2	4.68 ± 0.05^Bc^	6.10 ± 0.03^Cb^	8.04 ± 0.01^Ca^
T3	4.78 ± 0.02^Bc^	6.08 ± 0.01^Cb^	8.03 ± 0.02^Ca^
T4	4.71 ± 0.03^Bc^	5.99 ± 0.03^Db^	7.31 ± 0.05^Da^

^A–C^Means with different superscript in the same column significantly differ at *p* < 0.05

^a–c^Means with different superscript in the same row significantly differ at *p* < 0.05

^1^C-control, T1-injection with 10 mM sodium phosphate buffer (*pH* = 7.0) at 10 %, T2-injection with 10 mM sodium phosphate buffer (*pH* = 7.0) and 0.01 % BHT at 10 %, T3-injection with 10 mM sodium phosphate buffer (*pH* = 7.0) and 5 % plasma protein at 10 %, and T4-injection with 10 mM sodium phosphate buffer (*pH* = 7.0) and 5 % hydrolysis plasma protein at 10 %

### Protein solubility

Sarcoplasmic protein and total protein solubility values of samples treated with plasma protein were significantly higher than those of samples injected with the control and other treatment solutions (*p* < 0.05; Table 5). Sarcoplasmic protein denaturation may function as a more effective indicator of muscle quality (especially color) [41]. Changes in sarcoplasmic protein solubility have also been observed to influence WHC, shear force values, gel formation, and the emulsifying capacity of meat [42, 43]. It is also generally accepted that sarcoplasmic proteins are denatured at temperatures over 40 °C, during various heat treatments [41]. Thus, meat protein solubility is influenced by processing conditions, water content, salt content, heat applications, and changes in pH [42].

**Table 5:** Effects of injection with plasma protein solution on protein solubility (mg/g) in porcine *longissimus* muscle

Treatments^1)^	Total protein	Sarcoplasmic protein	Myofibrillar protein
C	197.75 ± 10.87	67.52 ± 1.41^B^	130.23 ± 9.46^AB^
T1	208.38 ± 10.82	65.93 ± 0.60^B^	142.45 ± 11.43^AB^
T2	217.39 ± 13.52	63.15 ± 1.04^C^	154.23 ± 14.57^A^
T3	201.21 ± 2.38	78.80 ± 0.73^A^	122.41 ± 1.65^B^
T4	210.65 ± 4.51	67.83 ± 1.20^B^	142.81 ± 3.30^AB^

^A–C^Means with different superscripts in the same column significantly differ at *p* < 0.05

^1^C-control, T1-injection with 10 mM sodium phosphate buffer (*pH* = 7.0) at 10 %, T2-injection with 10 mM sodium phosphate buffer (*pH* = 7.0) and 0.01 % BHT at 10 %, T3-injection with 10 mM sodium phosphate buffer (*pH* = 7.0) and 5 % plasma protein at 10 %, and T4-injection with 10 mM sodium phosphate buffer (*pH* = 7.0) and 5 % hydrolysis plasma protein at 10 %

Kauffman et al*.* [29] suggested that the increase in protein solubility observed in their study was likely caused by a reduction of protein denaturation in muscles of halothane-sensitive pigs treated with sodium bicarbonate. The findings of Marta et al*.* [44] highlight the dependence of myofibrillar and sarcoplasmic protein solubilities on meat quality and NaCl concentration. Further, an increase in total protein solubility, including sarcoplasmic protein solubility, has been reported to decrease drip loss in pork [31].

### Shear force

During cold storage, the shear force of the sample treated with hydrolysis plasma proteins was significantly lower than those of samples treated with the control and experimental solutions (*p* < 0.05; Table 6). Tenderness is a major palatability characteristic of meat. In this study, hydrolysis plasma protein injection was effective in reducing shear force, which is a measure of the toughness of meat. The reduction in shear force can be attributed to the increased water content of the treated meat as well as weakening of its myofibrillar structure. It has been suggested that marination greately improves the tenderness of pork compared to other factors pertaining to the production (e.g. breed and feeding levels) and processing (e.g., chilling rate, hip suspension, and electrical stimulation) of the meat [7]. Lawrence et al*.* [45] and Baublits et al*.* [46] reported similar differences in the shear force values of *longissimus* and *triceps brachii* steaks which were either untreated and or injected with water. Their findings suggest that mechanical tenderization was not responsible for the improvements in tenderness. According to Yasui et al*.* [47] this tenderizing effect might be attributed to the fact that polyphosphates promote weakening of the myosin heads to actin cross-bridge, and thus promote dissociation of actomyosin. Our results also suggest that the increased tenderness observed in the treated meat samples is likely due to higher water content and weakened muscle structure.

**Table 6:** Effects of injection with plasma protein solution on Warner-Bratzler shear force (kg/cm^2^) in porcine *longissimus* muscle, during cold storage

Treatments^1)^	Storage (days)		
	1	3	7
C	2.09 ± 0.28^Aa^	1.61 ± 0.22^Cb^	2.36 ± 0.42^Ba^
T1	2.49 ± 0.61^A^	2.80 ± 0.25^A^	2.91 ± 0.48^A^
T2	2.61 ± 0.49^A^	2.74 ± 0.19^A^	2.53 ± 0.45^AB^
T3	2.25 ± 0.65^A^	2.38 ± 0.17^B^	2.39 ± 0.36^B^
T4	1.46 ± 0.46^B^	1.16 ± 0.19^D^	1.15 ± 0.20^C^

^A–D^Means with different superscripts in the same column significantly differ at *p* < 0.05

^a–b^Means with different superscripts in the same row significantly differ at *p* < 0.05

^1^C-control, T1-injection with 10 mM sodium phosphate buffer (*pH* = 7.0) at 10 %, T2-injection with 10 mM sodium phosphate buffer (*pH* = 7.0) and 0.01 % BHT at 10 %, T3-injection with 10 mM sodium phosphate buffer (*pH* = 7.0) and 5 % plasma protein at 10 %, and T4-injection with 10 mM sodium phosphate buffer (*pH* = 7.0) and 5 % hydrolysis plasma protein at 10 %

### Lipid oxidation

**Fig. 1:**
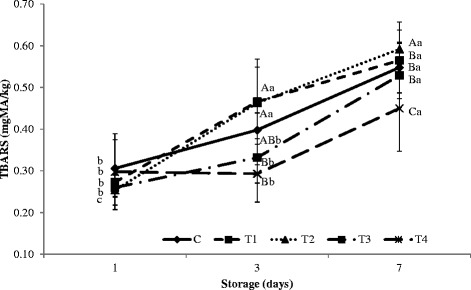
Effects of injection with plasma protein solution on TBARS in porcine *longissimus* muscle during cold storage

During cold storage, the interaction between storage period and type of treatment injection (non-hydrolysis and hydrolysis plasma protein solutions) had a significant effect on TBARS in the porcine *longissimus* muscle (*p* < 0.05; Fig. 1). While there were no differences in TBARS between samples at the beginning of cold storage, samples injected with hydrolysis plasma proteins showed significantly lower TBARS than samples injected with the control or other treatments after 7 days (*p* < 0.05). This decrease in TBARS indicates decreased oxidation during cold storage resulting from hydrolysis plasma protein treatment. The lower TBARS in the porcine *longissimus* muscle injected with hydrolysis plasma proteins may also be due to the antioxidant characteristics of milk powder [48, 49].

Hydrolysis and non-hydrolysis plasma protein solutions showed increased antioxidant activities during cold storage. According to Faustman and Cassens [32], lipid oxidation and myoglobin oxidation are closely related in meat an increase in one result in a similar increase in the other. This pattern was thought to be related to the direct oxidation of Mb or the destruction of Mb-reducing systems by free radicals generated during lipid oxidation. Guo et al*.* [50] reported that, in their study, the low molecular weight fraction (<1 k) of protease N hydrolysate of royal jelly proteins had the greatest antioxidant activity. Further, Park et al*.* [51] reported strong antioxidant activity in hydrolysate from egg yolk protein.

## Conclusions

Proximate composition of the injection of pre-rigor porcine *longissimus lumborum* muscle with non-hydrolysis and hydrolysis plasma protein showed higher moisture and lower protein contents than the control. The pH and lightness showed no differences between control and T4, whereas significantly higher redness and yellowness were found in T4. Hydrolysis plasma protein significantly decreased MetMb content during cold storage. Shear force was significantly lower in T4 than control and other treatments. The lower TBARS was observed in the porcine longissimus muscle injected with non-hydrolysis and hydrolysis plasma protein solutions compared to the control.

The main findings of our study are that (1) injection of pre-rigor porcine *longissimus lumborum* muscle with non-hydrolysis and hydrolysis plasma proteins improves pork quality and (2) *longissimus lumborum* muscle pH, meat color, tenderness, protein solubility, and lipid oxidation in pork loin are directly affected by the concentrations of injected non-hydrolysis and hydrolysis plasma proteins. Thus, our study clearly highlights the potential use of hydrolysis plasma proteins in improving the tenderness of pork and increasing storage life.
